# Whole genome characteristics of hedgehog coronaviruses from Poland and analysis of the evolution of the Spike protein for its interspecies transmission potential

**DOI:** 10.1186/s12917-024-04277-4

**Published:** 2024-09-21

**Authors:** Katarzyna Domanska-Blicharz, Anna Lisowska, Justyna Opolska, Jakub J. Ruszkowski, Maciej Gogulski, Małgorzata Pomorska-Mól

**Affiliations:** 1 Department of Poultry Diseases, National Veterinary Research Institute, al. Partyzantów 57, Puławy, 24-100 Poland; 2 Department of Animal Anatomy, University of Life Sciences in Poznań, ul. Wojska Polskiego 71C, Poznań, 60-625 Poland; 3 University Centre for Veterinary Medicine, University of Life Sciences in Poznań, Szydłowska 43, Poznań, 60-656 Poland; 4 Department of Preclinical Sciences and Infectious Diseases, University of Life Sciences in Poznań, ul. Wołyńska 35, Poznań, 60-637 Poland

**Keywords:** Hedgehog, Betacoronavirus, Poland, Whole genome, S gene

## Abstract

**Background:**

The hedgehogs have been recently identified as possible reservoir of Middle East respiratory syndrome coronavirus like (MERS-CoV-like). These viruses were classified as a distinct *Betacoronavirus erinacei* (BCoV-Eri) species within the *MerBCoV-Eriirus* subgenus. As coronaviruses are known for their ability to jump between different hosts, including humans, this can pose a particular threat to people in direct contact with hedgehogs, such as those working at animal asylums. Our previous studies have shown the presence of BCoV-Eri strains in animals collected in the wildlife rehabilitation centre. This study aimed to investigate the presence of CoV in subsequent hedgehogs collected from the urban area of Poland and their molecular characteristics.

**Results:**

Monitoring for the presence of coronavirus infection in hedgehogs revealed five positive individuals. The presence of BCoV-Eri was found in a total of 20% of animals tested. Our analyses revealed no correlation between CoVs positivity and animal health conditions but a higher probability of such infection in juveniles and females. The whole genome of two Polish *Hedgehog coronavirus 1* strains were sequenced and compared with available counterparts from European and Asian countries. Phylogenetic analysis showed that both CoV strains formed common cluster with other similar *MerBCoV-Eriirus*, but they were also found to be genetically variable and most changes in the S protein were identified. Our analysis revealed that some S protein sites of the *Hedgehog coronavirus 1* strains evolved under positive selection pressure and of five such sites, three are in the S1 region while the other two in the S2 region of the Spike.

**Conclusions:**

BCoV-Eri is to some extent prevalent in wildlife asylums in Poland. Given that the S protein of BCoVs-Eri is highly variable and that some sites of this protein evolve under positive selection pressure, these strains could potentially acquire a favourable feature for cross-species transmission. Consequently, the threat to humans working in such asylums is particularly high. Adequate biosecurity safeguards, but also human awareness of such risks, are therefore essential.

**Supplementary Information:**

The online version contains supplementary material available at 10.1186/s12917-024-04277-4.

## Background

The emerged in the 21st century three highly pathogenic betacoronaviruses for humans, namely severe acute respiratory syndrome coronavirus *(*SARS-CoV), SARS-CoV-2 and MERS-CoV are of zoonotic origin, as bat species have been established as their natural reservoir, although they have been shown to pass through so-called “intermediate” hosts as civets and raccoon dogs for SARS-CoVs and camels for MERS-CoV [1, 2]. Furthermore, recent studies clearly indicate that coronaviruses can cross species barriers and infect many other animals, undergoing adaptive evolutionary changes [3]. Efforts should therefore be made to uncover the diversity and abundance of viruses associated with wildlife and to assess which species have the greatest potential to transmit coronaviruses. Such an example is the coronaviruses identified in hedgehogs, which belong to the group of MERS-like coronaviruses, forming the distinct species *Betacoronavirus erinacei* within the subgenus *MerBCoV-Eriirus* (genus *Betacoronavirus*, family *Coronaviridae*, subfamily *Orthocoronavirinae*). The presence of these viruses has been identified in European hedgehogs (*Erinaceus europaeus*) but also in the Amur hedgehogs (*Erinaceus amurensis*) in China [4–9]. Thus, hedgehogs seem to be important reservoirs of MERS-like viruses and their possible role in the transmission of the virus from bats to humans may not be excluded.

*Betacoronavirus erinacei* genome is an approximately 30 kb long single-stranded, positive-sense RNA showing common ORFs reported for other *MerBCoV-Eriirus* strains. It consists of 16 nonstructural proteins (nsp1–16) encoded by open reading frame (ORF) 1a/b at the 5’end followed by the structural proteins spike (S), envelope (E), membrane (M), and nucleocapsid (N) as well as several auxiliary proteins encoded by other ORFs at the 3’end [6, 7]. On the other hand, in several strains detected in *Erinaceus europaeus* species in Italy an additional ORF CD200 ortholog was identified [7]. The coronaviral S glycoprotein is post-translationally cleaved into two functionally distinct domains: the N-terminal region (the S1 subunit) and the C-terminal part (the S2 subunit). The S1 plays an essential role in determining host ranges and tissue tropism and is further divided into the N-terminal domain (S1-NTD) and the C-terminal domain (S1-CTD), either of which can function as the receptor-binding domain (RBD) to induce cellular entry. The S2 fuses the membranes of the virus and host cells and contains the Fusion peptide, two Heptad repeats (1 and 2) and the Tans-membrane domain [10]. The viruses from the *MerBCoV-Eriirus* subgenus as HCoV-HKU4 and MERS-CoV recognize specific hDPP4 receptor through the S1-CTD [11]. However, analysis of this part of the S1 subunit of *Betacoronavirus erinacei* revealed many altered amino acids and also changed spatial conformations, which rather prevent the recognition of this hDPP4 receptor [7].

In previous studies, our group has investigated rectal swabs from 40 hedgehogs originating from the urban area of the Poznan city, Poland, collected in the Wildlife Rehabilitation Centre (WRC) during six months of 2020 (August-December) and found that 25% of them were betacoronavirus (BCoVs) positive [12]. Phylogenetic analysis based on the short fragment of viral replicase gene showed that all Polish BCoVs-Eri grouped together with other similar viruses identified in Western Europe. The purpose of this study was to continue the monitoring of coronaviruses in hedgehogs collected in during following few months 2021 and phylogenetic analysis of the detected viruses [12]. We also reported the whole genome characteristics of two merBCoV-Eriiruses identified in Polish hedgehogs. We have also attempted to determine whether the detected changes in protein S have been subjected to positive selection and whether they may have an impact on a possible host switch from non-human animals to humans.

## Results

### Hedgehog coronavirus 1 prevalence

Twenty-five adult (73.5%) and 9 (26.5%) juvenile animals were included in the study. Half the animals were females (17/34) and half were males (17/34). The mean body weight of juvenile animals was 218.22 ± 114.45 (range 75–426 g), while in the adult group it reached 648.32 ± 97.28 (504–850 g). Of 34 hedgehogs analysed, 5 were found to be CoV-positive (Additional file 1). In investigated group the CoV prevalence was 14.7% (95% CI: 6.45–30.13). 60% of positive hedgehogs were juveniles, while 40% were adults. Females represented 3 out of 5 positive animals (60%). Eleven animals were included to the group of sick animals (injured, diagnosed with fractures, weakening or with severe ectoparasite infestation). There was no significant relationship between clinical status, gender and age and detection of hedgehog coronaviruses (Table 1).

**Table 1:** Analysis of risk factors associated with BCoVs-Eri detection

Risk factor	Positive/tested^a^	% positive	*p*	OR (95% CI)
**Health status**			0.6399	1.534 (0.110-15.974)
sick	2/11	18.18		
clinically healthy	3/23	13.04		
**Gender**			1.0000	1.584 (0.1564–21.648)
female	3/17	17.64		
male	2/17	11.76		
**Age**			0.1023	5.3832 (0.500-78.428)
juvenile	3/9	33.33		
adult	2/25	8.00		

*p* value determined by two-sided Fisher’s exact test; *p* ≤ 0.05 considered significant. OR Odds ratio, 95% confidence interval. ^a^ Number of positive animals of all tested

The odds ratio calculated for gender and health status indicates approximately 1.5 times higher likelihood of having positive results in females compared to the males and in clinically healthy than in sick animals. After controlling for age, the odds of being bCoV RNA positive was over 5 times higher in juvenile animals (95% CI 0.500-78.428).

Phylogenetic analysis of the viral replicase gene fragment of five Polish strains obtained by the method used to detect coronaviruses showed that they grouped together with previously identified Polish and other European representatives of *Hedgehog coronavirus 1* species [12]. Homology between studied five Polish sequences was 95.6–99.2% and previously detected was 95.0-99.9%. They possessed the highest similarity with the viruses from Italian (95.9–98.7%) and German (93.7–97.4%) animals. The nt homology of Polish hedgehog CoVs to strains detected in Great Britain and China were 89.5–93.3% and 80.5–85.7%, respectively.

### NGS, sequence assembly and genome organization

As a result of NGS attempts, nearly whole genomic sequence was obtained for two samples out of 5 all processed. In the case of the hedgehog sample designated as J24, the NGS produced 2,965,121 read pairs specific for CoV, which were assembled into one contig of 29,743 nt in length with an average coverage of 13,855 reads. In turn, in the case of the hedgehog sample of J64, the NGS produced 2,547,260 read pairs specific for CoV, which were assembled into one contig of 30,195 nt in length with an average coverage of 12,878 reads. The complete genomes of viruses received in this study were named bCoV/Erinaceus/Poland/J24/2020 (abbreviated as J24/2020) and bCoV/Erinaceus/Poland/J64/2021 (J64/2021) and deposited to the GenBank under accession numbers PP721361-62.

The genomes organization of both Polish stains were similar to other European BCoVs-Eri and encoded in 11 open reading frames (ORFs) with the order: 5’UTR-1ab-S-3a-3b-4a-4b-5-E-M-8b-N-3′UTR. As shown in Table 2, the lengths of some regions/genes and their putative products were the same, while others were different. The ORFs/proteins with a constant conservative amount of nt and amino acids (aa) were replicase polyprotein, all structural and some accessory proteins: Rep1ab (21485 nt/7097 aa), S (3984 nt/1327 aa), 4a (246 nt/81 aa), 4b (627 nt/223 aa), E (249 nt/82 aa), M (657 nt/218 aa), 8b (564 nt/187 aa), and N (1278 nt/425 aa). Different lengths were ORFs encoding 3a, 3b and 5 proteins (303–315 nt/100–104 aa, 423–435 nt/140–144 aa, and 669–690 nt/222–229 aa, respectively).

**Table 2:** Regions/genes positions and lengths (nt and aa) of the J24/2020 and J64/2020 strains (structures differing in length are marked with a grey shadow)

Region/gene	5’UTR	Rep1ab	S	3a	3b	4a	4b	5	E	M	8b	*N*	3’UTR	Strain
Position	1-122	123-21607	21525-25508	25526-25828	255961-26030	25785-26030	26017-26688	266983-27387	27461-27709	27724-28380	28480-29043	28434-29711	29712-29743	**J24/2020**
Length (nt)	121	21,485	3984	303	435	246	672	690	249	657	564	1278	31	
Length (aa)	-	7097	1327	100	144	81	223	229	82	218	187	425	-	
Position	1-228	229-21713	21631-25614	25632-25946	25726-26148	25903-26149	26135-26806	26816-27484	27558-27806	27821-28477	28577-29140	28531-29808	29809-30195	**J64/2021**
Length (nt)	228	21,485	3984	315	423	246	672	669	249	657	564	1278	386	
Length (aa)	-	7097	1327	104	140	81	223	222	82	218	187	425	-	

**Fig. 1:**
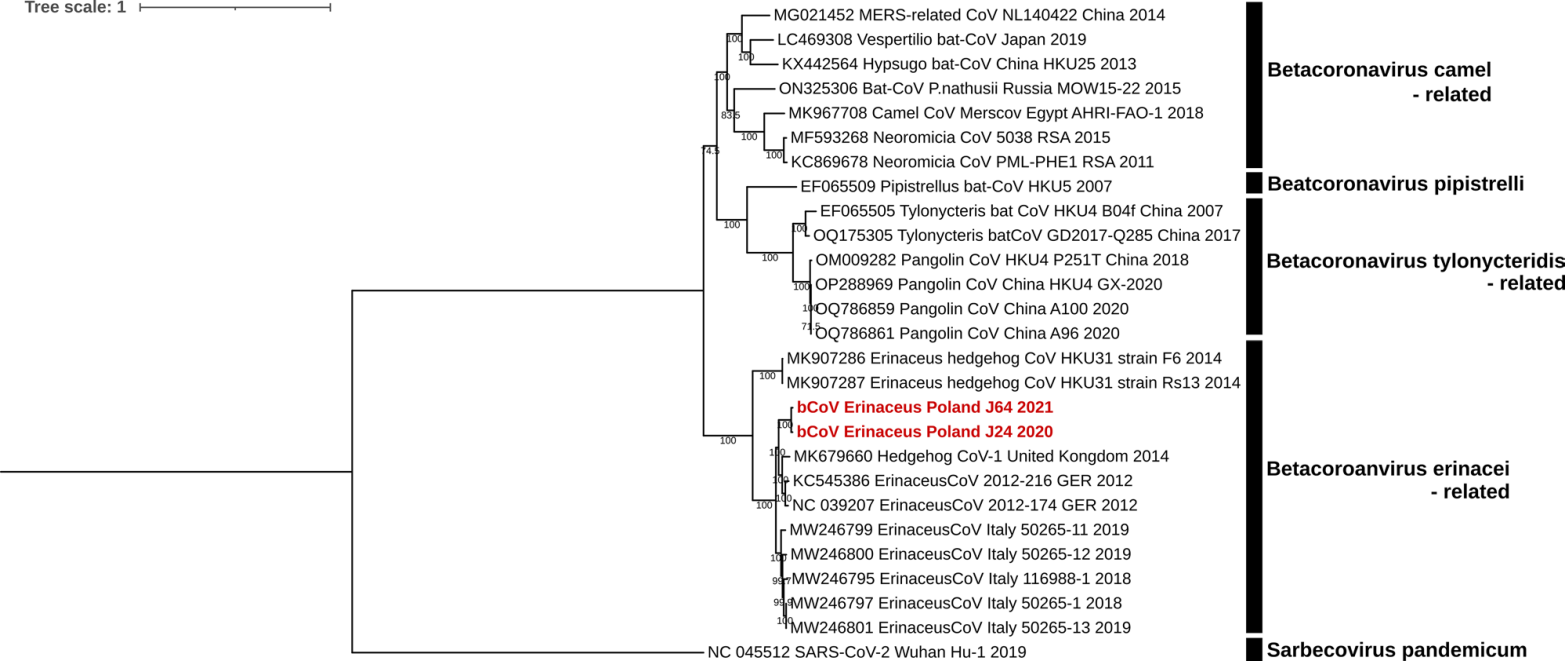
Phylogenetic tree of BCoVs-Eri based on the whole genomes. The tree was generated via IQ-TREE ver. 1.6.12 using the maximum likelihood analysis based on GTR + F + I + G4 model and 1000 bootstrap replicates (bootstrap values shown on the tree). SARS-CoV-2 used as the outgroup. Polish hedgehog BCoVs-Eri marked in red bold

The phylogeny based on the complete genome sequence revealed that the both Polish strains formed a common branch with other bCoV detected in hedgehogs (Fig. 1). Sequence analysis showed that they shared nucleotide identities of 98.4% at the complete genome level. The identities with respective HhCoVs from Germany, Italy and United Kingdom were 92.2–92.4%, 89.6–91.1% and 91.5–91.7%, respectively. Whole-genome homology to non-European hedgehog coronaviruses was much lower, at 79.3–79.4% (Additional file 2). When structural protein genes are considered, the lowest homology of 89.5–91.1% was found for the S gene. The homology of the other structural protein genes was higher, above 94%. Nucleotides homology between Polish strains was 97.6% for S gene, 98% for E and M genes and 98.7% for N gene. A similar tree topology was obtained in the case of the phylogeny of the amino acid sequences of the S protein (Additional file 3). Amino acids homology between Polish BCoVs-Eri was 96.8% and to the rest of European counterparts between 89.6 and 91.1%. However, comparison of the S1 protein results in 95.7% homology (88.0–90.0% to the European BCoVs-Eri) and S2 protein – 98.1% homology (91.1–92.7% to the European BCoVs-Eri). The selection pressure profiles of the S protein of Polish and other European BCoV-Eri strains were also analysed. As expected, the calculated dN/dS ratio was less than 1 (0,143), clearly indicating that the S protein of these strains evolved under negative selection. However, five individual codons were found under positive selection, four of them were indicated by three methods (MEME, FUBAR and FEL) and one, at the position 131 by two methods (MEME and FUBAR). These codons were located both in the S1-NTD (three of them) as well as in the S2 protein (two of them). These residues were in the following positions: 27 (G/D/S/F/V), 131 (P/T/I/K/Q), 222 (T/N/D), 866 (V/G/S/N) and 1272 (Y/H) (p-value < 0.1). Of these five sites, three are located in the S1-NTD region, while the other two are in the S2 region, one located around the Fusion peptide region and the other just downstream of the Heptad 2 repeat.

A comparison of the receptor binding domain (RBD) of the Polish strains and the other European and the Asian CoVs detected in hedgehogs is shown in Table 3 (and Additional file 4). Among the 18 amino acid residues of MERS-CoV known as individually connected with either van der Waals contacts or H-bond or salt-bridge interactions with different residues in type II transmembrane protein, hDPP4, four (in the positions Y490, D524, D526 and Y527) in Polish virus strains were found. On the other hand, the other eleven amino acids of this region in all coronaviruses identified in European hedgehogs remain unchanged. However, it should be stressed that in this short, 83-aa long region, the similarity between European BCoVs-Eri is 80.7–90.9%, while European to Asian HKU13 from the Amur hedgehog is 63.9–73.5%. Despite the detected degree of variability in this region, no change is unique to Polish BCoVs-Eri.

**Table 3:** Alignment of key amino acids sequences in the receptor binding domain of the spike protein. Residues critical themselves or responsible for critical bond formation are labelled with (*)/(**). Residues conserved among analysed sequences are highlighted in bold

			**	*	*	*	**		**		*	*		**		*	*	*
MERS-CoV	**D** **455**	**P** **463**	**Y** **499**	N 501	**K** **502**	**L** **506**	D 510	**R** **511**	**E** **513**	**W** **535**	**E** **536**	**D** **537**	**G** **538**	**D** **539**	**Y** **540**	R 542	W 553	**V** **555**
HKU4	Y 460	N 468	**Y** **503**	S 505	**K** **506**	**L** **510**	N 514	D 516	**E** **518**	S 540	**E** **541**	**D** **542**	**G** **543**	Q 544	V 545	K 547	L 558	I 560
HKU31	T 443	A 452	**Y** **491**	S 493	R 494	T 498	-	-	-	S 523	N 524	**D** **525**	V 526	**D** **527**	**Y** **528**	G 530	F 538	I 540
Polish BCoVs-Eri	T 443	A 451	**Y** **490**	S 492	R 493	I 497	**-**	**-**	**-**	P 523	S 523	**D** **524**	A 525	**D** **526**	**Y** **527**	G 529	Y 537	L 539
Italian BCoVs-Eri	T 444	A 452	**Y** **491**	S 493	R 494	T/I 498	-	-	-	K/P/Q 523	K/S/N 524	**D** **525**	A/V 526	**D** **527**	**Y** **528**	G 530	Y 538	**V/**L **540**
German BCoVs-Eri	T 443	A 451	**Y** **491**	S 493	R 494	V 497	-	-	-	P 522	S 523	**D** **524**	A 525	**D** **526**	**Y** **527**	G 529	Y 537	L 539
UK BCoV-Eri	T 442	A 450	**Y** **489**	S 491	R 492	T 496	-	-	-	P 521	S 522	**D** **523**	A 524	**D** **525**	**Y** **526**	G 528	Y 536	L 538

The methods used to analyze recombination event did not demonstrate its existence in the genomes of Polish coronaviruses.

## Discussion

In the present study, we continued the moniotoring of coronavirus infections in hedgehogs delivered to the WRC in the Polish city of Poznań. In our previous hedgehog surveillance, we detected 10 positive hedgehogs out of 40 tested [12]. In turn, another 34 hedgehogs were delivered for testing over the next few months and five of them were infected with betacoronavirus. The presence of hedgehog coronavirus was found in a total of 20% (15/74) of the hedgehogs tested. Such BCoV-Eri prevalence appears to be lower compared to the prevalence in hedgehogs in other countries [4–7]. On the other hand, as stated previously, the presence of detected viruses in these animals may depend on many factors, such as sampling season, sample handling, the detection method used but also a living model (hibernate stage) [12]. And although in most studies they have been identified in hedgehogs with clinical signs of disease, recent reports from Italy but also from China tend to indicate that these betacoronaviruses are more likely to be commensals in the hedgehog body [9, 13, 14]. Our previous analyses also revealed no correlation between CoVs positivity and animal health conditions and additional studies of hedgehogs presented here support this findings. In addition, the results presented showed a higher probability of BCoV-Eri infection in juveniles and females and these results are also in agreement with our previous results, with age-dependence showing a stronger relationship and sex a slightly lower relationship than in previous analyses [12]. However, it should be added that performed statistical analysis was done on a rather small sample of data; the results obtained would need to be confirmed on more data. So far, information on the whole genome characterization of European BCoV-Eri strains is not abundant [4, 6, 7]. Moreover, recent paper brings rather surprising information on the presence of an additional ORF CD200 ortholog in some Italian strains [7]. It was even suggested that some CoVs can acquire host genes potentially involved in the immune-modulatory cascade and possibly enabling the virus to escape the host defense. Therefore, studies have been undertaken on the molecular characterization of the whole genome of BCoVs-Eri circulating in the hedgehog population in Poland.

Whole genome sequences of Polish BCoVs-Eri exhibited features of other European BCoVs-Eri sharing with them 89.6–92.4% nucleotide identity but homology to non-European hedgehog coronaviruses was much lower, at 79.3–79.4%. No other structures as the CD200 orthologue previously found in Italian strains of BCoV-Eri were detected in the genomes analyzed. Among structural protein genes, the lowest homology of 89.5–91.1% was found for the S gene, although a similar tree topology was obtained for the whole genome phylogeny as well as for the S gene. Nevertheless, a thorough analysis of the this structure was undertaken, i.e. analysis of the receptor-binding region as well as the selection pressure profile of the Polish and all other known BCoVs-Eri.

The Polish BCoVs-Eri RBD had only 4 conserved amino acids of the 18 such known necessary for MERS-CoV binding to the human hDPP4 receptor [2]. A similar amino acid sequence was identified in Italian, German or British coronaviruses [4, 6, 7]. In addition, examination of Italian coronaviruses revealed the absence of the spatial structures necessary for such interaction, suggesting that these viruses have no affinity for human receptors [7]. On the other hand, the use of the hDPP4 receptor by HKU4 bat coronavirus has been demonstrated, despite having three more conserved amino acids than the BCoV-Eri strains in comparison to MERS-CoV (a total of 7 conserved amino acids out of 18 ones of MERS-CoV) [2].

Nevertheless, the same amino acids for receptor binding at the RBD are conserved in all European coronaviruses and also some in Asian hedgehogs, implying an affinity for the same receptor in hedgehog body tissues.

Our analysis showed also that the BCoV-Eri strains were evolving under negative selection pressure but some sites of S protein are positively selected, and of the five such sites, 3 are located in S1-NTD region. Previous studies on the adaptive evolution of the main five genes, namely orf1ab, S, E, M and N in the four genera of coronaviruses indicated that purifying selection is dominant evolutionary force driving coronavirus evolution [10, 15]. However, S genes and particularly the S1 coding region revealed faster evolution than non-S genes. More positively selected amino acid sites in S1-NTD than S1-CTD regions were also previously identified suggesting that other factors besides the receptor-binding region found in betacoronaviruses in the S1-CTD are involved in positive selection [10]. It is generally accepted that S1-NTD mainly recognizes host cell sugar receptors to facilitate virus entry, although in some coronaviruses it can also bind to protein receptors. Therefore, the positive selection signal identified in S1-NTD may reflect continuous attempts by coronaviruses to adapt to a different host species or tissue [16]. In addition, this part of the S1 region could also potentially be an epitope that induces neutralizing antibodies in the host [10]. The humoral immune response in hedgehog may be weaker than in other animals taking into account that they enter a hibernation period of several months, during which their metabolism slows down considerably [17]. Despite these facts, changes in S1-NTD region may reflect the escape of the coronavirus from the host immune response. We also found two signals of positive selection within the S2 region, one located around Fusion peptide region and the next one just after the Heptad repeat 2. The changes in the Fusions peptide region may increase cell to cell fusion activity and change in the virulence of the virion as seen in mutant studies of SARS-CoV and SARS-CoV-2 [18, 19]. In turn, the Heptad repeat 2 region was previously thought to determine host expansions and therefore, facilitate virus cross-species transmission [20].

## Conclusion

In conclusion, the evidence for the circulation of merBCoV-Eriiruses in Polish urban hedgehogs was further supported. Monitoring of hedgehogs during 18 months period showed a 20% prevalence of these infections. The analyses presented here showed no correlation between positive CoVs and the presence of clinical signs of infection. The identified hedgehog coronaviruses were related in terms of the whole genome structure to other European BCoVs-Eri. However, they were found to be genetically variable based on the sequence analysis described here, and most changes were found in the S protein, although the same amino acids for receptor binding are conserved in all European coronaviruses and also some in Asian hedgehogs, suggesting an affinity for the same receptor in hedgehog body tissues. Our analysis also showed that BCoV-Eri strains evolved under negative selection pressure, but that some S-protein sites are positively selected, and of five such sites, three are in the S1-NTD region while the other two are in the S2 region. Some of these sites may allow the acquisition of favorable feature for cross-species transmission.

## Methods

### Hedgehogs sampling

Rectal swabs were collected from 34 hedgehogs found in the city of Poznan, Wielkopolskie Voivodeship, Poland. As previously described, these animals were brought to the WRC because they were sick, injured or too young to survive on their own. Once delivered, they were subjected to a thorough visual inspection, sampled using swabs with transport medium (UTM^®^:Viral Transport, COPAN, USA) and given appropriate treatment if necessary. The samples were collected over the course of 8 months from the following number of animals: April 2021 (*n* = 6), June (*n* = 11), July (*n* = 3), October (*n* = 3), and November (*n* = 11).

### Coronavirus detection

After extraction of total RNA from the obtained fluids using RNeasy Mini kit (Qiagen, Germany), the presence of CoV was detected using a RT-PCR assay in a nested configuration. The first step (RNA transcription and DNA amplification) with the Qiagen OneStep RT-PCR kit (Hilden, Germany) and the second step (nested DNA amplification) with the Platinum™ Taq DNA Polymerase kit (Invitrogen, Carlsbad, USA) were performed. In all reactions primers containing several degenerate nucleotides useful for amplification of the viral replicase gene fragment of all known mammalian and avian coronaviruses were used [21]. Obtained products were visualized after electrophoresis and observed as characteristic bands of 555 bp in case of the CoV presence (detailed protocols available on request). Sequencing were done in both directions using Sanger technology in commercial service (Genomed Sp. z o.o., Warsaw, Poland).

### Whole genome sequencing

In order to obtain the complete genome sequences of the samples identified as CoV-positive in the present and previous studies (15 positives), selected samples with the highest viral genome content (strongest amplicon obtained by PCR) were subjected to next-generation sequencing (NGS) using the Genomed Sp. z o.o. (Warsaw, Poland) commercial service. A total of five coronavirus field samples were subjected to NGS. Briefly, samples (rectal swabs) were treated with TURBO DNase (Life Technologies, USA) and RNase One (Promega, USA) to remove DNA and extracapsid RNA. The isolation of viral RNA from such treated samples was carried out and then retrotranscribed into DNA using a Superscript IV First-Strand cDNA Synthesis Kit (Invitrogen, USA) and the second strand was synthesized with the addition of Klenow polymerase (New England Biolabs, USA). A 150 bp long paired-end DNA library was prepared using a Nextera XT sample preparation kit (Illumina Inc) and sequencing was performed using a MiSeq Reagent kit v3 (Illumina Inc).

### Genome sequence assembly, analysis and phylogeny

Quality control of obtained raw reads was carried out using fastp software. The reads were deduplicated *via* Picard. Sequences were mapped to MW246798 (ErinaceusCoV/Italy/50265-17/2018), MK679660 (Hedgehog coronavirus 1) and KC545386 (ErinaceusCoV/2012 − 216/GER/2012) by Geneious Prime software, v2023.0.4 (Biomatters Ltd., New Zealand). The forward and reverse nucleotide sequences resulted from Sanger technology were edited and aligned in the final consensus also using the same Geneious software. The obtained nucleotide sequences were then compared among themselves and then with other sequences from the GenBank database using the BLAST algorithm, and those with the highest homology were extracted for further analysis (http://www.ncbi.nlm.nih.gov/BLAST/). Alignments of nucleotide sequences were performed using the MAFFT method and the percentage of nucleotide and amino acid sequences similarities were assessed in mentioned Geneious Prime software. The alignments were then exported to IQ-TREE ver. 1.6.12 software to perform maximum likelihood phylogenetic analysis using the best-fitting substitutions [22]. Bootstrap analyses of the resultant trees were performed using 1000 replicates. The tree visualization was performed using the iTOL v6 online tool [23].

To detect any recombination events, the complete genome of the detected CoVs, and selected the most similar sequences were analyzed using different methods available in the RDP package v.4 [24]. Only recombination events identified by at least three different methods with p-value below 1.0 × 10E-10 were taken into account.

To check if individual codon sites in the whole S gene of European BCoV-Eri strains are subjected to positive or purifying selection pressure, an analysis was carried out using various bioinformatics tools of the Hy-Phy package (www.datamonkey.org). The ratio of non-synonymous (dN) to synonymous (dS) nucleotide substitutions per site (dN/dS) and the selection pressures using methods for individual codons were estimated (Fixed-Effects Likelihood – FEL, Fast Unconstrained Bayesian Approximation – FUBAR and Mixed Effects Model of Evolution - MEME) [25–28]. Positively selected sites were only these confirmed by at least two different method.

Additionally, the nucleotide sequence of the S gene was also deduced into amino acids and analyzed for the presence of key residues for binding to a potential receptor.

### Statistical analyses

The associations between CoV RNA detection in samples, demographic features (species, gender, age), and health status variables were estimated using Fisher’s exact test. The Wilson method for small n was used to calculate a 95% confidence interval (95% CI) for CoV RNA prevalence. All statistical analyses were performed in Statistica13.3 software (Tibco, USA).

## Electronic supplementary material

Below is the link to the electronic supplementary material.

Supplementary Material 1: Additional file 1 (.doc, Demographic information and health status of hedgehogs included in the study, table with different data as delivery date, sex, age, body weight, clinical condition, CoV status of hedgehogs studied)

Supplementary Material 2: Additional file 2 (.doc, Sequence identity of the complete genome and individual genes and proteins of bCoV/Erinaceus/Poland/J24/2020 and bCoV/Erinaceus/Poland/J64/2021 to other betacoronavirus strains, table with values)

Supplementary Material 3: Additional file 3 (.doc, Phylogenetic tree of European and Asian hedgehogs BCoVs-Eri based on the Spike protein. The tree was generated via Q-TREE ver. 1.6.12 using the maximum likelihood analysis based on JTTDCMut+I+G4 model and 1000 bootstrap replicates (bootstrap values shown on the tree). Polish hedgehog BCoVs-Eri marked in red bold)

Supplementary Material 4: Additional file 4 (.doc, Alignment of multiple sequences of the receptor binding domain (RBD) showing variation in key amino acids. Critical residues and critical bond formation residues are contained in red boxes. Numbering above alignment confers to the MERS-CoV sequence)

## Data Availability

The datasets generated and/or analysed during the current study are available in the NCBI repository [accession numbers PP721361-62] as well as in the Datamonkey archives: [http://datamonkey.org/meme/65d88521f17fd757eef44f7d, http://datamonkey.org/fubar/65d88718f17fd757eef45460, http://datamonkey.org/slac/65d888b8f17fd757eef45612, http://datamonkey.org/fel/65d88e25f17fd757eef4578a, http://datamonkey.org/gard/65d89322f17fd757eef458e9].

## Abbreviations

CoV: Coronavirus
BcoV: Betacoronavirus
MERS-CoV: Middle East Respiratory Syndrome Coronavirus
BCoV-Eri: Betacoronavirus Erinacei
SARS-CoV: Severe Acute Respiratory Syndrome Coronavirus
SARS-CoV-2: Severe Acute Respiratory Syndrome Coronavirus 2
