# Supplementary material for: Whole genome characteristics of hedgehog coronaviruses from Poland and analysis of the evolution of the Spike protein for its interspecies transmission potential
Source: BMC Vet Res. 2024 Sep 21;20:424. doi: 10.1186/s12917-024-04277-4 (PMC11415979; doi:10.1186/s12917-024-04277-4)
Supplement: Supplementary file 1 — Supplementary Material 1: Additional file 1 (.doc, Demographic information and health status of hedgehogs included in the study, table with different data as delivery date, sex, age, body weight, clinical condition, CoV status of hedgehogs studied) [file 12917_2024_4277_MOESM1_ESM.docx]

**Additional file 1** Demographic information and health status of hedgehogs included in the study

| **delivery date** | **sex** | **age** | **body weight**  **(g)** | **clinical condition on admission, diagnosis** | **CoV status** | **GenBank number** |  |
| --- | --- | --- | --- | --- | --- | --- | --- |
| 04.2021 | F | ad | 640 | injury, fractured skull | - |  | |
| 04.2021 | M | ad | 662 | kidney injury, vomiting | - |  | |
| 04.2021 | M | ad | 650 | injury to fingers and wrists | - |  | |
| 04.2021 | F | ad | 650 | weak, died up to 6h after admission | - |  | |
| 04.2021 | F | ad | 640 | clinically healthy | - |  | |
| 04.2021 | M | ad | 527 | weak, died up to 1h after admission | - |  | |
| 06.2021 | M | ad | 640 | fracture of the skull, euthanasia | - |  | |
| 06.2021 | M | ad | 842 | weak | + | PP721357 | |
| 06.2021 | M | ad | 721 | clinically healthy | - |  | |
| 06.2021 | M | ad | 550 | clinically healthy | - |  | |
| 06.2021 | M | ad | 850 | clinically healthy, slightly weakened | - |  | |
| 06.2021 | M | ad | 713 | clinically healthy, slightly weakened | - |  | |
| 06.2021 | F | ad | 510 | Eye wound, bloody nose discharge | - |  | |
| 06.2021 | F | juv | 75 | clinically healthy | - |  | |
| 06.2021 | M | juv | 80 | clinically healthy | - |  | |
| 06.2021 | M | juv | 164 | clinically healthy | - |  | |
| 06.2021 | M | ad | 670 | clinically healthy | - |  | |
| 07.2021 | F | ad | 691 | clinically healthy | - |  | |
| 07.2021 | M | ad | 504 | clinically healthy | - |  | |
| 07.2021 | F | ad | 550 | Wounded, wound myiasis | - |  | |
| 10.2021 | F | juv | 196 | clinically healthy | + | PP721358 | |
| 10.2021 | F | juv | 220 | Wounded, wound myiasis | + | PP721362 | |
| 10.2021 | F | juv | 347 | Severe dyspnoea | - |  | |
| 11.2021 | F | ad | 659 | clinically healthy | - |  | |
| 11.2021 | M | ad | 535 | clinically healthy | - |  | |
| 11.2021 | F | ad | 740 | clinically healthy | - |  | |
| 11.2021 | M | ad | 798 | clinically healthy | - |  | |
| 11.2021 | F | ad | 689 | clinically healthy | - |  | |
| 11.2021 | F | juv | 198 | clinically healthy | + | PP721359 | |
| 11.2021 | M | ad | 531 | clinically healthy | + | PP721360 | |
| 11.2021 | M | ad | 657 | clinically healthy | - |  | |
| 11.2021 | F | juv | 426 | clinically healthy | - |  | |
| 11.2021 | F | ad | 589 | clinically healthy | - |  | |
| 11.2021 | F | juv | 258 | clinically healthy | - |  | |

ad – adult, juv – juvenile; + CoV-positive individuals; - CoV-negative individuals
