# Supplementary material for: Whole genome characteristics of hedgehog coronaviruses from Poland and analysis of the evolution of the Spike protein for its interspecies transmission potential
Source: BMC Vet Res. 2024 Sep 21;20:424. doi: 10.1186/s12917-024-04277-4 (PMC11415979; doi:10.1186/s12917-024-04277-4)
Supplement: Supplementary file 2 — Supplementary Material 2: Additional file 2 (.doc, Sequence identity of the complete genome and individual genes and proteins of bCoV/Erinaceus/Poland/J24/2020 and bCoV/Erinaceus/Poland/J64/2021 to other betacoronavirus strains, table with values) [file 12917_2024_4277_MOESM2_ESM.docx]

**Additional file 2** Sequence identity of the complete genome and individual genes and proteins of bCoV/Erinaceus/Poland/J24/2020 and bCoV/Erinaceus/Poland/J64/2021 to other betacoronavirus strains (in bold - the highest nt identity; in bold italic - aa identity)

|  | **Genome** | **RdRp** | | **S** | | **E** | | **M** | | **N** | |
| --- | --- | --- | --- | --- | --- | --- | --- | --- | --- | --- | --- |
|  |  | **nt** | **aa** | **nt** | **aa** | **nt** | **aa** | **nt** | **aa** | **nt** | **aa** |
| **bCoV/Erinaceus/Poland/J24/2020** | | | | | | | | | | | |
| bCoV/Erinaceus/Poland/J64/2021 |  |  |  | 97.6 | 96.8 | 98.0 | 95.1 | 98.0 | 97.7 | 98.7 | 99.1 |
| NC 039207 ErinaceusCoV/2012-174/GER/2012 | 92.4 | **93.9** | ***93.5*** | ***90.3*** | ***90.9*** | **96.0** | 96.3 | **95.1** | *96.8* | 92.4 | 96.5 |
| KC545386 ErinaceusCoV/2012-216/GER/2012 | 92.5 | 93.8 | ***93.5*** | 90.1 | 90.6 | **96.0** | 96.3 | **95.1** | 96.8 | **92.9** | ***96.5*** |
| MK679660 Hedgehog CoV-1 United Kingdom/2014 | 91.7 | 93.0 | 92.8 | 89.3 | 89.8 | 95.6 | ***97.6*** | 94.5 | ***98.2*** | 92.2 | 96.0 |
| MW246800 ErinaceusCoV/Italy/50265-12/2019 | 90.1 | 91.2 | 90.7 | 89.0 | 89.5 | 94.0 | 95.1 | 93.5 | 95.0 | 91.0 | 95.1 |
| MW246801 ErinaceusCoV/Italy/50265-13/2019 | 89.5 | 91.2 | 90.7 | 88.9 | 90.4 | 95.2 | 95.1 | 93.8 | 95.8 | 90.8 | 94.8 |
| MW246797 ErinaceusCoV/Italy/50265-1/2018 | 89.5 | 91.2 | 90.8 | 88.9 | 90.5 | 95.2 | 95.1 | 93.8 | 95.4 | 90.8 | 94.8 |
| MW246795 ErinaceusCoV/Italy/116988-1/2018 | 91.1 | 91.4 | 91.0 | 88.7 | 89.7 | 94.8 | 96.3 | 93.8 | 95.4 | 90.3 | 94.6 |
| MW246799 ErinaceusCoV/Italy/50265-11/2019 | 89.3 | 90.8 | 90.6 | 88.1 | 89.8 | 95.2 | 96.2 | 93.5 | 95.0 | 91.1 | 95.8 |
| MK907287 Erinaceus hedgehog CoV HKU31 strain Rs13 | 79.3 | 80.2 | 79.7 | 78.1 | 78.0 | 88.7 | 84.1 | 87.6 | 92.2 | 81.4 | 86.4 |
| MK907286 Erinaceus hedgehog CoV HKU31 strain F6 | 79.3 | 80.2 | 79.7 | 78.1 | 78.0 | 88.7 | 84.1 | 87.6 | 92.2 | 80.2 | 83.6 |
| **bCoV/Erinaceus/Poland/J64/2021** | | | | | | | | | | | |
| NC 039207 ErinaceusCoV/2012-174/GER/2012 | 92.7 | **93.4** | ***95.3*** | ***90.2*** | ***91.1*** | **96.8** | 95.1 | **96.0** | *96.8* | 91.9 | 95.1 |
| KC545386 ErinaceusCoV/2012-216/GER/2012 | 92.6 | 93.1 | ***95.3*** | 90.1 | 90.9 | **96.8** | 95.1 | **96.0** | 96.3 | **92.4** | ***95.8*** |
| MK679660 Hedgehog CoV-1 United Kingdom/2014 | 91.9 | 92.6 | 92.6 | 89.3 | 90.2 | 96.0 | ***97.6*** | 94.8 | ***97.2*** | 91.7 | 95.3 |
| MW246800 ErinaceusCoV/Italy/50265-12/2019 | 92.4 | 91.3 | 90.0 | 89.1 | 90.3 | 93.6 | 96.3 | 94.4 | 96.3 | 90.1 | 95.1 |
| MW246801 ErinaceusCoV/Italy/50265-13/2019 | 92.4 | 93.2 | 90.8 | 89.1 | 90.2 | 94.8 | 96.3 | 94.7 | 95.9 | 89.4 | 94.1 |
| MW246797 ErinaceusCoV/Italy/50265-1/2018 | 92.4 | 93.2 | 90.8 | 89.1 | 90.4 | 94.8 | 96.3 | 94.7 | 95.9 | 89.4 | 94.1 |
| MW246795 ErinaceusCoV/Italy/116988-1/2018 | 92.2 | 93.3 | 91.0 | 88.9 | 90.2 | 94.4 | 96.3 | 94.7 | 95.9 | 89.0 | 94.4 |
| MW246799 ErinaceusCoV/Italy/50265-11/2019 | 92.0 | 92.9 | 90.6 | 88.1 | 89.6 | 94.8 | 96.3 | 94.7 | 96.3 | 90.1 | 95.3 |
| MK907287 Erinaceus hedgehog CoV HKU31 strain Rs13 | 82.0 | 83.4 | 73.0 | 78.4 | 78.3 | 85.9 | 84.1 | 86.6 | 92.2 | 82.0 | 85.9 |
| MK907286 Erinaceus hedgehog CoV HKU31 strain F6 | 81.9 | 83.4 | 73.0 | 78.4 | 78.2 | 85.9 | 84.1 | 86.6 | 92.2 | 81.8 | 83.1 |
