# Supplementary material for: Whole genome characteristics of hedgehog coronaviruses from Poland and analysis of the evolution of the Spike protein for its interspecies transmission potential
Source: BMC Vet Res. 2024 Sep 21;20:424. doi: 10.1186/s12917-024-04277-4 (PMC11415979; doi:10.1186/s12917-024-04277-4)
Supplement: Supplementary file 3 — Supplementary Material 3: Additional file 3 (.doc, Phylogenetic tree of European and Asian hedgehogs BCoVs-Eri based on the Spike protein. The tree was generated via Q-TREE ver. 1.6.12 using the maximum likelihood analysis based on JTTDCMut+I+G4 model and 1000 bootstrap replicates (bootstrap values shown on the tree). Polish hedgehog BCoVs-Eri marked in red bold) [file 12917_2024_4277_MOESM3_ESM.docx]

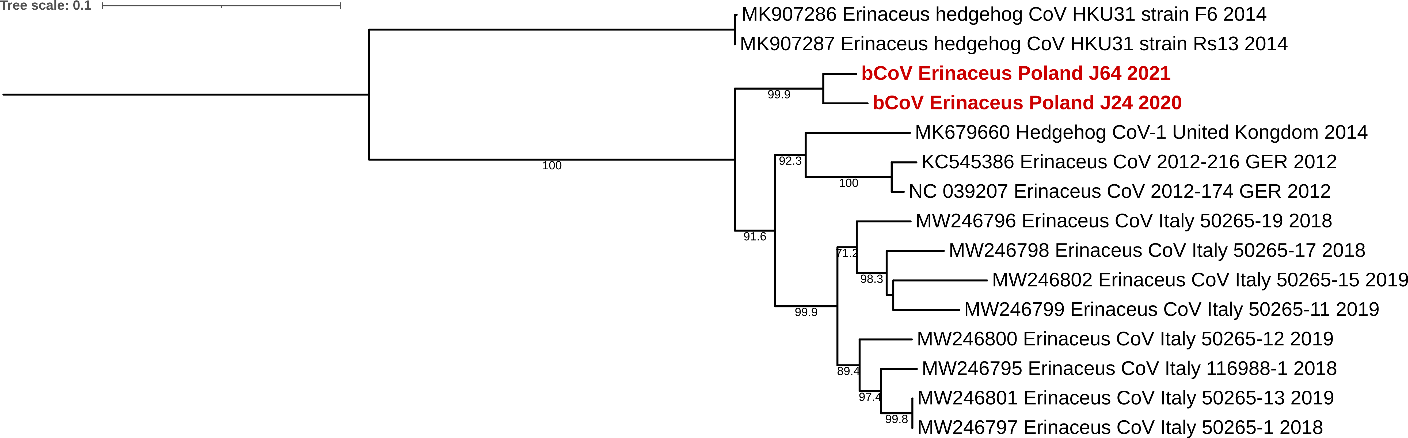


**Additional file 3** Phylogenetic tree of European and Asian hedgehogs BCoVs-Eri based on the Spike protein. The tree was generated via Q-TREE ver. 1.6.12 using the maximum likelihood analysis based on JTTDCMut+I+G4 model and 1000 bootstrap replicates (bootstrap values shown on the tree). Polish hedgehog BCoVs-Eri marked in red bold.
