# Supplementary material for: Whole genome characteristics of hedgehog coronaviruses from Poland and analysis of the evolution of the Spike protein for its interspecies transmission potential
Source: BMC Vet Res. 2024 Sep 21;20:424. doi: 10.1186/s12917-024-04277-4 (PMC11415979; doi:10.1186/s12917-024-04277-4)
Supplement: Supplementary file 4 — Supplementary Material 4: Additional file 4 (.doc, Alignment of multiple sequences of the receptor binding domain (RBD) showing variation in key amino acids. Critical residues and critical bond formation residues are contained in red boxes. Numbering above alignment confers to the MERS-CoV sequence) [file 12917_2024_4277_MOESM4_ESM.docx]

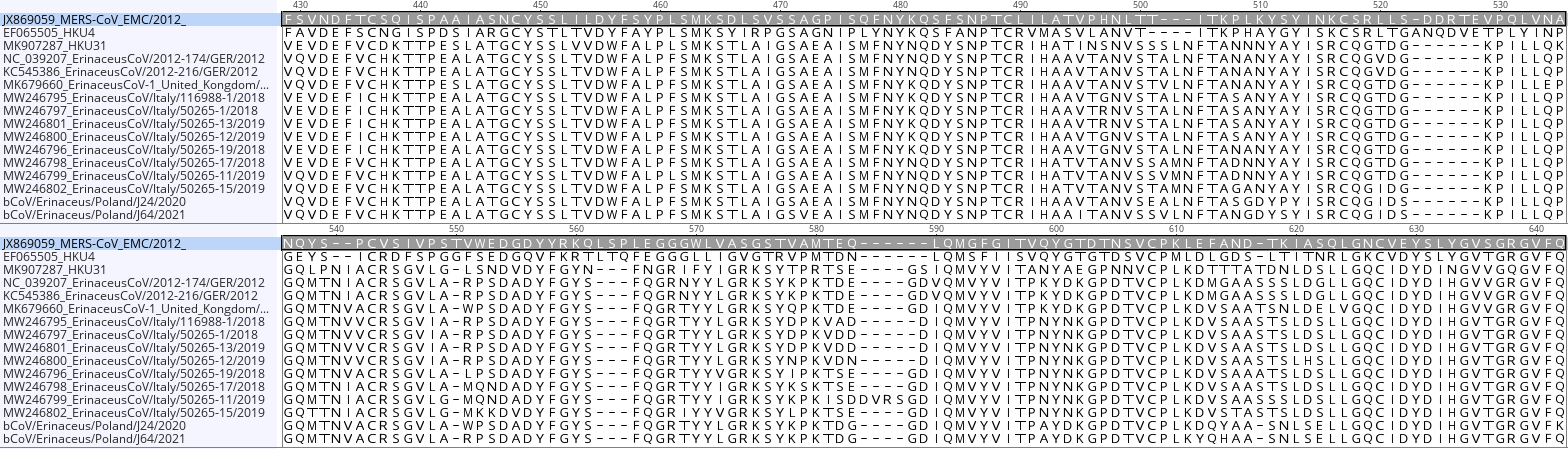


**Additional file 4** Alignment of multiple sequences of the receptor binding domain (RBD) showing variation in key amino acids. Critical residues and critical bond formation residues are contained in red boxes. Numbering above alignment confers to the MERS-CoV sequence
